# Report to the Transport Board on the Subject of Yellow Fever
*It is not incumbent on us to state the sources from whence we occasionally derive official documents of this kind; but we pledge ourselves that the documents are authentic; and that is enough. We have reason to know, that the author of this paper (who we hope will excuse our giving publicity to it without its coming from himself) is the elegant writer of the Article—"Medical Topography of New Orleans" in the 12th Vol. of the Edinburgh Medical and Surgical Journal. This is a sufficient reason for our estimation of any thing from the same pen. Editors.


**Published:** 1817-07

**Authors:** Archibald Robertson


### *Report to the Transport Board on the Subject of Yellow Fever.**

H. M. S. Cydnus, Portsmouth,
*January*, 1816.

IN my Journal for last year, I stated it as my belief, that the Endemic Fever of the West Indies was certainly not contagious. In taking pains to state the evidence on that point, which my experience had furnished, I was almost inclined to think, that I had employed myself superfluously in re-discussing a question, which had been already decided by a body of evidence, amounting to apparent certainty; evidence indeed, so various, as few contested points in medicine can boast. The united learning, diligence, and sagacity of former observers, had pronounced the disease non-infectious; and the correctness of the induction seemed to be supported by the subsequent experience of every hour.

From being abroad at the time, I was unacquainted with the news of the medical world; and was ignorant of the weight which the recent works of Dr. Pym and Sir James Fellowes have thrown into the opposite scale; and I will own, I read, on my return home, with a good deal of sur-

---

* It is not incumbent on us to state the sources from whence we occasionally derive official documents of this kind; but we pledge ourselves that the documents are authentic; and that is enough. We have reason to know, that the author of this paper (who we hope will excuse our giving publicity to it without its coming from himself) is the elegant writer of the Article—"MEDICAL TOPOGRAPHY OF NEW ORLEANS" in the 12th Vol. of the Edinburgh Medical and Surgical Journal. This is a sufficient reason for our estimation of any thing from the same pen. EDITORS.

prise, those tenets of theirs, which are so hostile to the generally-adopted opinion of medical men where I had lately been. Fraught, as they are, with proofs apparently unobjectionable, and ushered into the world under the auspices of their names, their talents, and their rank, it was to be expected that the books would produce an universal impression on the opinions of the profession, and of the public.

There are two tenets maintained by them, so interesting in a scientific point of view, and so important to the welfare of the community, that they may be said to contain the very *heart's-core* of the question. These tenets are, 1st. That the fever in question, spreads from person to person by contagion; and, 2dly, That one attack of the disease, be it sustained in the West Indies or elsewhere, grants a full immunity against all subsequent attacks.

It is by facts, and not by assertions, that the public mind will at last be guided; I shall, therefore, bring forward some conspicuous instances bearing pointedly on the tenets alluded to; but before doing so, a few general remarks are necessary for clearing the avenues to the question, so as to prevent the embarrassment arising from extraneous matter, as well as the possibility of being misunderstood.

In the first place, on the subject of the identity of the Gibraltar Fever with that of the West Indies, Dr. Pym has left abundant room for interminable cavilling, and all about mere words! Either the diseases are the same, or they are different. Similarity of phænomena and of character, if not indubitable tests of identity, are, at least, the only ones that our faculties can appreciate. Since then the similiarity of both fevers is complete, why distinguish that of Gibraltar by so many different names? Why fritter it away into tiresome and unprofitable subdivisions, until the diagnosis becomes difficult in theory, and impossible in practice? Why increase an already burthensome system of nosology, and introduce greater complexity into the more than sufficiently difficult subject of fevers? In all diseases, single cases are not alike severe; and even in the West Indies, where the fever is wont to be seen in its direct forms, of four cases that occur, the *first*, perhaps, may have febrile symptoms, which, when early attended to, will be removed by open bowels, quietude, and low diet. In the *second*, they will perhaps be more severe, and require both bleeding and purging; and on the third day, the patient will have a remission of all the symptoms. In the

*third* case, the fever will be more violent still, and distinguished by congestion in the brain, and great irritability of stomach ; so that the patient, though liberally bled and purged, will suffer severely from what are called the secondary symptoms of fever, and yet recover at last. The case of the *fourth* shall be distinguished by great violence, all along ; and the early occurrence of bad symptoms ; in spite of every resource he will die on the second, third, or fourth day, with signs of yellowness and high putrescency (as it is called). These four forms, though greatly differing in their phænomena and their result, no medical man denies to be merely accidental varieties of the same fever ; and that treatment or other adventitious circumstances will often make the *first* and *second* pass rapidly into the character of the *third* and *fourth,* or vice versâ. If this system is to be adopted of *splitting down* diseases, we shall shortly have nearly as many new names of fever, as there are individual cases !

The *modus operandi* of contagion, as well as its essence, are removed from the cognizance of our external senses ; and therefore, like all other occult and impalpable causes, are not the subjects on which demonstrative certainty is to be obtained. I am persuaded, that many fevers are attributed to contagion, in which it had no share whatever, merely because, when men find it difficult to explain the cause of disease, their impatience prompts them to get rid of the embarrassment at once, by calling in contagion, which always affords an easy and popular solution of the difficulty, if not a philosophical one. To be sure, this is, at best, but *cutting* the Gordian knot which we cannot untie ; and to assume the operation of a power which, perhaps, was not present. " Nisi dignus vindice nodus," is an axiom of more than poetical utility, and ought to be well remembered in the present case ; for the question of contagion or non-contagion is not a curious speculation, calculated to whet the edge of controversy and exercise the subtlety of medical scholars, but is a matter of vital importance to the political and domestic condition of mankind, as well as to the cause of science.

I am of opinion, that fever has often been ascribed to contagion (supposed to be generated, no one knows how) when miasmata were the real and only cause. The latter are certainly the most widely diffused, and the most universal cause of fever, throughout the whole domain of Nature. Their agency does not seem limited to any determinately short time ; they are imbibed now, and probably do not excite their peculiar effects for weeks to come; though latent, they are not idle. They are operating their purpose unseen, unfelt, and unknown. The patient, during this deceitful interval, feels little or no retrocession from health or abatement of spirits. Like the unhappy URIAH, he carries about with him, unconsciously, the cause and instrument of his own destruction! He perhaps, at most, looks pale and sallow; but the change will be remarked by others sooner than himself. At last, actual disease commences, and then staggers us with the difficulty of accounting for it.

To prove this lurking property of miasmal poison, and the indeterminate time of its operation, proofs are by no means wanting. The first is the notorious fact, that nine-tenths of the fevers that appear on board ships, in a tropical climate, do not appear immediately on the application of the cause, but in the first fourteen days or three weeks after putting to sea: of this fact I, in common with other naval surgeons, have had abundant evidence, as my Journals will shew.*

I shall here mention a circumstance which bears upon the point now touched on.—During August and September, 1815, the Cydnus cruized off the Capes of Virginia and around Bermuda. The heat was very great, yet not a single instance of fever occurred. This was to us a singular exemption: for after leaving any West Indian port,

* I was, at first, at a loss to account for this fact: the following considerations seem to explain it. In tropical climates, a ship usually comes into harbour from a cruize in as good health as if she had been serving in the European seas. The stay in port is always as brief as possible, seldom more than six, and never exceeding nine or ten days. During this time, the men are as seldom as possible allowed to go on shore, and pains are taken to keep them sober. They are sheltered from the sun and night-dews by means of awnings, and the service seldom requires them to be exposed in any considerable degree. But on proceeding to sea, a portion of the crew are unavoidably exposed to the direct rays of the sun, by their duties in the tops, &c. At night, also, when the awnings are removed, men, during their watch on deck, are exposed to the night air, and can seldom be altogether prevented from sleeping any-where they can, exposed to the dews. But besides these general causes, it seems next to certain, as I have above stated, that the miasma which causes endemic fever (unless when imbibed in doses of unusual strength or virulence) lies latent for an indefinite number of days in the system, without producing any perceptible derangement therein, until it is stimulated into activity by certain accessory or occasional causes. That exposure to the sun's heat and sleeping in the night dews are amongst the most powerful of such causes, I need not waste time in proving.

and even Bermuda, we had never missed having a few cases of fever; but, in the present instance, we had none, because we had sailed from Halifax, where the heat of the atmosphere is not sufficient to raise and give activity to miasmal exhalations; and where, of course, none could have been imbibed by the people. This seems to me the only supposeable reason: for where we were then cruizing, the thermometer was always about eighty, which is nearly the mean temperature of the West Indies; and from the air being loaded with moisture, the heat was very oppressive to the sensations. The fact just stated is an indirect and unexpected proof of the topical origin of the ardent fever of southern latitudes—that it owes its origin to local miasmata, and that it is by these miasmata, and not by contagion, that cases of it are continued and disseminated.

In the second place, ships leaving the West Indies often lose men by fever on the passage home, (even though they have got into the temperate latitudes) and have men taken ill with violent remittents, even after the ship's arrival in an English port. On this point, Dr. John Hunter's authority is very conclusive. Had the ship continued in a tropical temperature, instead of a remittent or intermittent, the men would have had ardent fever, *i. e.* the disease in its violent continued form.

In my Journal for the last year, there are detailed the cases of two boys seized with severe intermittents at Halifax, N. S. They had both been at Maranham (S. America), where ardent fever was prevailing epidemically: there they must have imbibed miasmata *four weeks* prior to their being taken ill; for, in the interim, they had been on board a healthy frigate at sea, without once coming nigh land. There is also in the Journal, the case of one of the crew of the Cydnus, who was taken ill at Halifax with ardent fever; of which he must have imbibed the cause nearly a month before at Bermuda or Havannah. In the interim, he had not been out of the ship; contagion was out of the question, as no case of fever had existed in the ship for a length of time previous to his becoming ill: besides, in him the symptoms were too severe to be merely sporadic, or the result of intemperance.

With such instances of the irregular power of miasmata on the human frame, who shall define the point where they cannot operate; and where contagion must necessarily rank as a cause?

Respecting the proofs that have occurred to me personally, of the non-contagiousness of this fever, I have nothing material to add to what was noted in the general remarks to my former Journal.* But of the mistaken opinion that one attack of the disease is a complete security against a second, I have had some striking and pointed refutations.

When his Majesty's ship Cydnus was sent to the West Indies, it was expected, on grounds of popular belief, by several of the officers, that as most of the crew had been previously inured to hot climates, by long servitude in the East and West Indies and Mediterranean, (in the Tribune, Cornelia, Sir Francis Drake, and Invincible, the ships from which they had been drafted into ours,) they would enjoy, *this time*, an unusual share of good health. But, alas! it was the very reverse; for the sick-book and journals exhibit a list of diseases almost unprecedented in a ship of her class. Let us mark what took place in regard to second attacks of fever. In July 1814, a man *(Hawkey)* had a most violent fever on board the *Cydnus*, who had a similar one two years before, while in the *Tribune*, on the Barbadoes station. The same may be said of *Parker*, who had before had a similar fever at Tarragona or Carthagena, in Spain. In August, 1814, *M'Donald* died on board the Cydnus of fever; he had it two years before in the *Tribune*. In September, *Pritchard* had the endemic, and recovered from it; he had had a similar fever some years before, on board the Cornelia. In November, 1814, *Howard* had fever of the first grade, from which he happily recovered; he had a second attack in March 1815, (after an interval of perfect health) and died. In August, 1814, *Ridgely* had the ardent fever, and recovered; in May, 1815, he had a second most severe attack, and recovered then also.

* The following observation is what was there noted:—"While men have been labouring under this fever, I have invariably adopted the practice of making the messmates of the sick man visit and sit with him, lift him in and out of bed, administer his drink, and the like, from observing that these attentions were more acceptable and cheering to the patient, than when bestowed by persons less immediately his companions. In such offices, it is evident that these men must be exposed to contagion in all manner of ways, if it exists. Yet, notwithstanding, not one in the same mess with the person labouring under fever, nor any of the ordinary attendants on the sick berth, were ever seized with the disease. So far, therefore, as negative evidence can go, this is conclusive; and in this matter, negative evidence is the only sort we can obtain. When indefinitely accumulated, the degree of certainty it produces, is tantamount to positive evidence."

I shall sum up the above evidence in a table, at the end of these remarks.

The foregoing evidence is unexceptionable, because it can be authenticated by a reference to my Journal; and it was collected and noted at a time when I had not the most distant idea that any question like the present would ever be agitated.

Finally, in all my conversations with professional men abroad, I never met one who believed yellow fever to be contagious, or who would not have resisted the imputation to him of such an opinion, as derogating from his character as a medical scholar. Nor have I met any one who believed that one attack of the disease operated as a preservative against a second, upon any other principle than that the disease itself, and the active evacuations necessary for its cure, diminish the rigidity of the fibre, and lessen the tone of the whole system in such a manner, that the unassimilated stranger becomes, in future, less liable to this, as well as to all other diseases of increased action.

Upon the whole, therefore, I think I may say that, however the abettors of contagion may support their system, they will have to support it in the face of the testimony of an enlightened body of men, who, for numbers and unanimity, are unequalled in the annals of medical controversy.

TABLE, *exhibiting a List of Men who, to my knowledge, have had more than one attack of Endemic or Ardent Fever.*

| Men's Names and Quality. | Place and Date of First Attack. | In what Ship at that time employed. | Place and Date of Second Attack. | In what Ship then employed. | Remarks. Whether Died or Recovered. |
|---|---|---|---|---|---|
| Owen Hawkey, (alias Awkey) seaman. | Barbadoes station, 1812. | Tribune | Jamaica station 1814. | Cydnus | Recovered. |
| J. Parker, Gun-room Steward | Tarragona or Carthagena, 1813. | Malta | Jamaica station July, 1814. | Cydnus | Recovered. |
| John M'Donald, (alias M'Donnell) seaman. | Barbadoes station, 1812. | Tribune | Jamaica station Aug. 1814. | Cydnus | Died. |
| Wm. Pritchard, Quarter-master. | East Indies, year uncertain. | Cornelia | Jamaica station Sept. 1814. | Cydnus | Recovered. |
| Wm. Howard, seaman. | Jamaica station, Nov. 1814. | Cydnus | Havannah, March, 1815. | Cydnus | Died. |
| Dan. Ridgely, seaman. | Jamaica station, Aug. 1814. | Cydnus | Havannah, May, 1815. | Cydnus | Recovered. |

P. S. I firmly believe that this far-famed disease, primarily in itself, and in ordinary situations, is *not* contagious. Whether or not it may ultimately become so, from improper crowding, filth, and imperfect ventilation, I do not profess to give an opinion, never having seen the disease under these unfavourable circumstances.

(Signed) ARCHIBALD ROBERTSON.

